# Dislocation of temporo-mandibular joint - an uncommon circumstance of occurrence: vaginal delivery

**Published:** 2010-06-25

**Authors:** Abderrahim El Bouazzaoui, Smael Labib, Ali Derkaoui, Mohammed Adnane Berdai, Azzeddine Bendadi, Mustapha Harandou

**Affiliations:** 1 Department of Anesthesia and Resuscitation, Mother and Child Hospital, University Hospital Hassan II, Fez, Morocco

**Keywords:** Dislocation, temporo-mandibular joint, vaginal delivery, Morocco

## Abstract

Dislocation of temporo-mandibular joint (TMJ) is an infrequent disease but still spectacular. This disease consists of a permanent, to some extent complete disruption of the temporo-mandibular joint. These dislocations often occur in a context of yawning, and less frequently after a burst of laughing or relatively mild facial trauma (slap, punch on the chin). We report a case of TMJ occurring in an uncommon circumstance: vaginal delivery. A woman aged 24-years with no special past medical history; primipara was admitted in the Department of Maternity of the University Hospital Hassan II of Fez for an imminent delivery of a twin pregnancy. Ten minutes after admission, the patient delivered vaginally with episiotomy. She gave birth to twins weighing 2800g and 2400g. During labour, and due to efforts of crying, the patient developed a sudden and immediate loss of function of the temporo-mandibular joint, with difficulty of speaking, the mouth permanently opened and with the chin lowered and thrown forward. The examination found an empty glenoid fossa of the temporo-mandibular joint in both sides. The diagnosis of dislocation of the TMJ was established. A CT scan of facial bones was done, objectifying a bilateral dislocation of TMJ. The reduction of this dislocation was performed in the operating room under sedation.

## Background

Dislocation of temporo-mandibular joint (TMJ) is an infrequent disease but still almost spectacular. This disease consists of a permanent, to some extent complete disruption of the temporo-mandibular joint. These dislocations often occurs in a context of yawning, and less frequently after a burst of laughing or relatively mild facial trauma (slap, punch on the chin).We report a case of TMJ occurring in an uncommon circumstance: vaginal delivery.

## Patient and case report

A young woman aged 24-years with no special past medical history; primipara was admitted in the Department of Maternity of the University Hospital Hassan II of Fez for an imminent delivery of a twin pregnancy. Obstetrical analgesia was not possible so the parturient cried in a strong manner during labour. Ten minutes after admission, the patient delivered vaginally with episiotomy.

**Figure 1:**
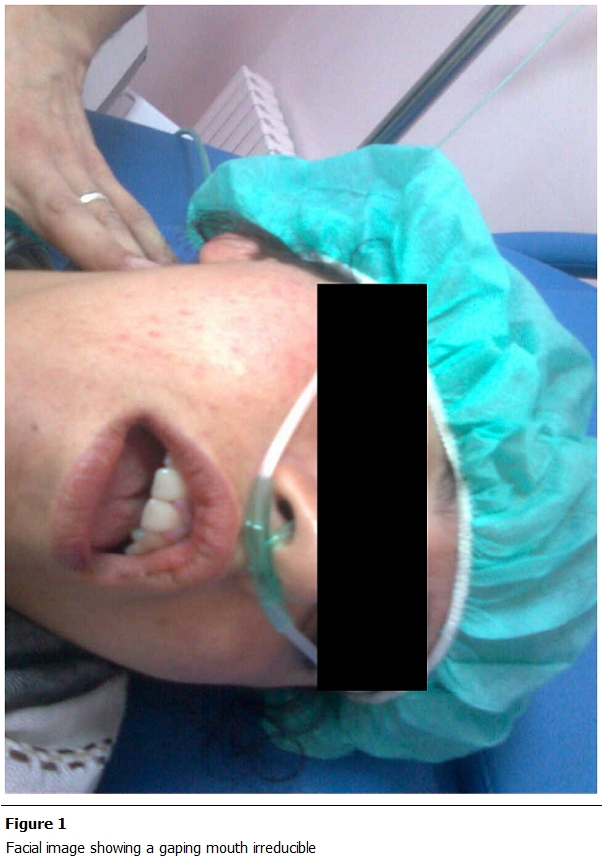
Facial image showing a gaping mouth irreducible

**Figure 2:**
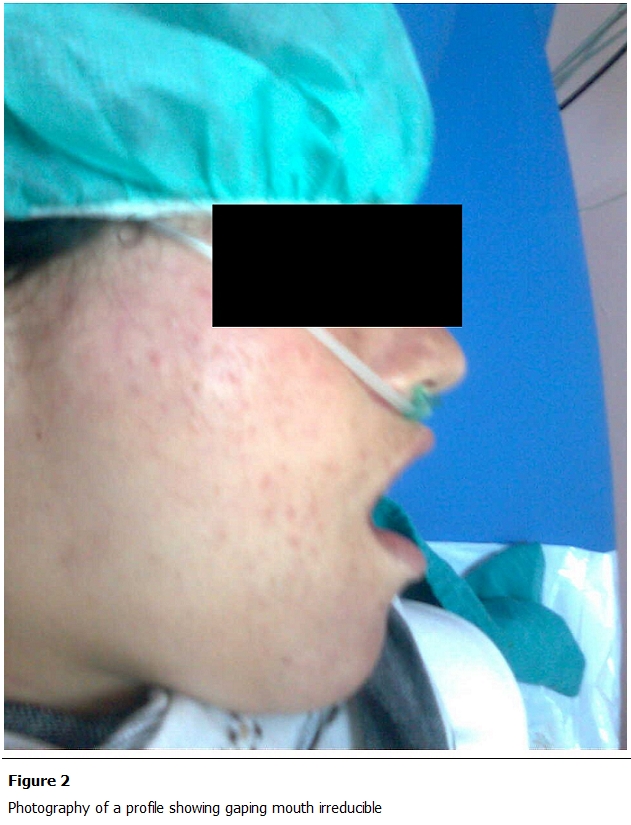
Photography of a profile showing gaping mouth irreducible

**Figure 3:**
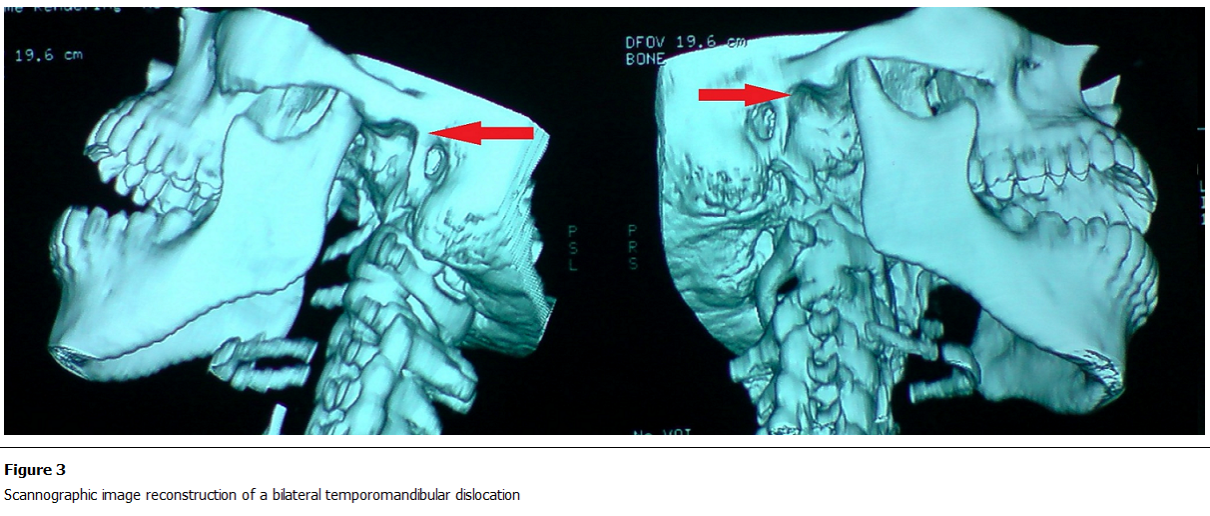
Scannographic image reconstruction of a bilateral temporomandibular dislocation

She gave birth to twins weighing 2800g and 2400g. During labour, the patient presented a sudden and immediate loss of function of the temporo-mandibular joint, with difficulty of speaking, the mouth permanently opened, with the chin lowered and thrown forward (Figure 1, 2). The examination found an empty glenoid fossa of the temporomandibular joint in both sides. The diagnosis of dislocation of the TMJ was established. Performance of special radiologic screening to study the TM was technically not possible. A CT scan of facial bones was performed, objectifying a bilateral dislocation of TMJ (Figure 3). The reduction of this dislocation was performed in the operating room under sedation.

## Discussion

The temporo-mandibular joint (TMJ), the only movable joint of the massive cranio-facial structure, helps to unite the base of the skull to the mandible. Temporo-mandibular joint dislocation is defined as the loss, permanent and more or less completely, of the normal anatomical relationships between the mandibular condyles and temporal [1]. Diseases related to the TMJ concern about a 1/3 of the general population [2].

The dislocation of the TMJ although a relatively uncommon disease, remains spectacular. Risk factors predisposing to these dislocations are known and include conditions such as dimorphism, algo-dysfunctional syndrome of the mastication apparatus, and past history of dislocation [3]. These dislocations occur most often after an effort of yawning or in cases of voluntary forced opening of the mouth [3]. Cases of spontaneous dislocation of the TMJ have been reported during sedation or in slow induction using propofol in patients at risk [4]. A female predominance is found in the literature, and would be linked, according to some authors; first to an ordinary ligament laxity because of hormonal impregnation and secondly to estrogen-progestin contraceptive treatment or alternative which would increase the likelihood of dysfunction of TMJ [5].

In our case, we report an uncommon circumstance of occurrence of TMJ dislocation, which to our knowledge, was never described in the literature; TMJ dislocation during a vaginal delivery (crying during uterine contractions). The examination in our patient did not reveal past history of dislocation of the TMJ and clinical examination did not find any facial dimorphism or hypermobility. The diagnosis of these dislocations is performed by clinical examination with the following main features: an open blocked mouth, disturbance of dental occlusion and emptiness of one or both temporal glenoid [3].

The manual reduction of the dislocation is an emergency. It must be performed as promptly as possible after the diagnosis is established [6,7]. Late diagnosis of this complication requires anesthesia to allow manual reduction [8]. In practice, there is no precise definition of  “a late diagnosed dislocation”; its diagnosis should lead to the attempt of a simple manual reduction. Only in the case of failure to reduce the dislocation manually that sedation in the operating room should be considered. In the extreme, a surgical approach to the joint could be attempted [3].

Once the diagnosis of dislocation is established, Nélaton maneuver must be performed. The operator places the pads of his thumbs on the molars of the patient with his fingers hooked around the mandibular angle. After obtaining a sufficient relaxation of the patient, the operator exerts a gentle and steady pressure directed downward and exaggerates mouth opening. It thus facilitates the maneuver by gently pushing the mandible backward to reintegrate the heads in the condylar glenoid. Immobilization of the mandible by bandage is then done [3].

## Conclusion

The dislocation of the TMJ is an uncommon disease; its occurrence during delivery is unusual. The Nélaton maneuver, well-known by ENT surgeons, must be known by any medical practitioner.

## Competing interests

The authors declare the have no conflicts of interest.

## Authors’ contribution

All authors contributed to the realization and description of this medical observation. The first and second authors managed the case of the patient during obstetrical anesthesia care.The third and fifth authors were responsible for the literature search. The first, second, fourth and the latter are responsible for the final drafting of medical observation.
